# Traditional Knowledge and Nutritive Value of Indigenous Foods in the Oraon Tribal Community of Jharkhand: An Exploratory Cross-sectional Study

**DOI:** 10.1080/03670244.2015.1017758

**Published:** 2015-04-22

**Authors:** Suparna Ghosh-Jerath, Archna Singh, Preeti Kamboj, Gail Goldberg, Melina S. Magsumbol

**Affiliations:** ^a^ Indian Institute of Public Health-Delhi, Public Health Foundation of India, Gurgaon, India; ^b^ Department of Biochemistry, All India Institute of Medical Sciences, New Delhi, India; ^c^ MRC Human Nutrition Research, Elsie Widdowson Laboratory, Cambridge, United Kingdom; ^d^ Public Health Foundation of India, New Delhi, India

**Keywords:** India, indigenous foods, medicinal plants, nutrient composition, nutrition security, Oraon

## Abstract

Traditional knowledge and nutritional value of indigenous foods of the Oraon tribal community in Jharkhand, India was explored. Focus group discussions were conducted with adult members to identify commonly consumed indigenous foods. Taxonomic classification and quantitative estimation of nutritive value were conducted in laboratories or utilized data from Indian food composition database. More than 130 varieties of indigenous foods were identified, many of which were rich sources of micronutrients like calcium, iron, vitamin A, and folic acid. Some were reported having medicinal properties. Utilization and ease of assimilation of indigenous foods into routine diets can be leveraged to address malnutrition in tribal communities.

The global community is increasingly looking towards finding means of sustainable nutrition for the growing population across all countries. The emphasis is on identifying low-resource strategies acceptable to communities that do not put an unnecessary burden on the environment (Food and Agriculture Organization 1993). One suggested approach is the adoption of an ecosystem approach in agricultural management with an emphasis on traditional and indigenous coping strategies. Traditional foods are those which indigenous peoples have access to locally, without having to purchase them, and within traditional knowledge and the natural environment from farming or wild harvesting (Kuhnlein, Erasmus, and Spigelski 2009). A synchronized plan for both the conservation and judicious utilization of food related resources involves working with indigenous communities to document traditional knowledge of edible plants, food processing, and medicinal value of local animal and plant resources. The tribal communities in India are a good example of indigenous populations with a vast cultural diversity, traditions, and environments (International Fund for Agricultural Development 2003; Singh, Singh, and Sureja 2007). There is a rich habitat of natural foods in Indian tribal environments that could possibly be used to promote food security, nutrition, and health. However, multiple factors including those related to geography, available agricultural technology, socio-cultural practices, and conditions in the community may lead to poor nutrition and health in these communities (Bhattacharjee et al. 2009).

According to the most recent Indian census, in Jharkhand, India, scheduled tribes constitute of 26.2% of the total population (Office of The Registrar General and Census Commissioner 2011). The Oraon are the second largest tribe in Jharkhand and also live in other states such as Chattisgarh, Bihar, West Bengal, and Odisha (Society of Tribal Women for Development 2014). They predominantly depend on agriculture for their livelihood along with some contribution from forestry, and labor with minor contribution from diverse occupations. Studies have reported sub-optimal nutritional status of children and adults of this community (Das and Bose 2012). The changing landscape in the background of deforestation and environmental degradation that is inevitable in an economy in transition like India (Hassan, Scholes, and Ash 2005) also presents a challenge to the maintenance of livelihoods, agricultural and environmental biodiversity. This has additional implications for finding sustainable community entrenched strategies to tackle malnutrition (Turner, Plotkin, and Kuhnlein 2013).

Hundreds of indigenous foods like plants, insects, and fungi worldwide are known to have food value (Boa 2004; DeFoliart 1992; Kuhnlein et al. 2009; Rathode 2009), but the nutrient content of many of these foods are undocumented and an assessment of the patterns of their intake is not available. Thus the present study was undertaken to explore the food environment of Oraon tribal community specifically with respect to use, nutritive value and traditional knowledge of indigenous foods. It involved listing, identification and taxonomic classification of indigenous foods, followed by nutrient composition analysis, if their nutritive values were not documented in the Indian Food Composition tables (Gopalan, Sastri, and Balasubramanian 1989).

## MATERIALS AND METHODS

### Study Design

This was an exploratory cross-sectional study conducted in villages of Jharkhand, India.

### Study Area

The study was conducted in four purposively selected villages inhabited by Oraon tribal community in Gumla district of Jharkhand. These were selected based on their geographic location, from a list of villages inhabited by the Oraon tribes, and included Nawagarh, Birkera, Sursang in Raidih Block and Guniya in Ghagra Block of Gumla district of Jharkhand. This work is part of a larger study that documented the role of indigenous foods in addressing nutritional and food security among indigenous communities in India. The research team included nutritionists, a qualitative researcher, and a biochemist. They were assisted by a field NGO active in these tribal districts. Multilingual speakers from the NGO well-versed in tribal dialects and also able to interact with the research team in English and Hindi were involved in the data collection. The fieldwork was conducted from March to November, 2013. This study was approved by the Institutional Ethics Committee of the Public Health Foundation of India. Informed consent was obtained from all the adult participants in the study by the data collection team; those who were literate gave signed consent forms. Verbal consents were documented in presence of a third-party witness.

#### Qualitative Methods

Qualitative methods described below were used for listing and eliciting preferences for commonly consumed indigenous foods.

#### Free listing and focus groups

Free listing and focus group discussions (FGDs) were used to assess the range of available foods and the contribution of indigenous wild foods to the regular diets of the Oraon community. Adult women and men and village elders were requested to join in FGDs. The research team started with a free listing exercise and explored various issues related to seasonal availability and access to these local foods. The participants identified indigenous or “*desi*” foods gathered from the local environment such as nearby forests (*jungle*), agricultural fields or bunds, gardens (*bari* or kitchen garden) or water resources such as man-made ponds (*pokhar*), dams or even from market *(haat*). The foods identified were categorized under various food groups based on their edible parts.

#### Pairwise ranking

This ranking method which compares pairs of elements helps prioritize preferences for needs, problems, food items etc and normally leads to analysis of the decision making rationale (Narayanasamy 2009). The participants followed this method and listed the most commonly consumed food items in a particular food group category. The food items were then tabulated as a matrix on a flip chart. Participants were then asked to compare the first food item in the row with various food items listed in the column one by one. The next step was to ask them to move on to the second food item in the row; keeping that as a constant and compare it with the third and the subsequent food items and enter the preference in the relevant grid. These steps were repeated till all the food items listed in the row were compared with the subsequent food items listed in the columns pairwise. A score was provided based on the number of times each food item was selected (Narayanasamy 2009). Thus, a hierarchy of series of food items in the various food groups were identified (table 1).

**TABLE 1:** Pairwise Ranking

	*Sarla saag*	*Saru saag*	Spinach	Radish leaves	*Munga saag*	*Phutkal saag*
*Sarla saag*	×	*Sarla saag*	Spinach	*Sarla saag*	*Munga saag*	*Phutkal saag*
*Saru saag*	*Sarla saag*	×	*Saru saag*	Radish leaves	*Munga saag*	*Phutkal saag*
Spinach	Spinach	*Saru saag*	×	Spinach	*Munga saag*	*Phutkal saag*
Radish leaves	*Sarla saag*	Radish leaves	Spinach	×	*Munga saag*	*Phutkal saag*
*Munga saag*	*Munga saag*	*Munga saag*	*Munga saag*	*Munga saag*	×	*Munga saag*
*Phutkal saag*	*Phutkal saag*	*Phutkal saag*	*Phutkal saag*	*Phutkal saag*	*Munga saag*	×

*Note. Munga saag –* 10; *Phutkal saag –* 8; *Sarla saag –* 4; Spinach *–* 4; *Saru saag –* 2; Radish leaves *–* 2.

### Quantitative Methods

The quantitative methodology used for identification and analysis of food items consumed by the community included:

#### Identification of food samples

Based on the free listing activity done through FGDs, a list of commonly consumed indigenous food items was compiled (including cereals, legumes, vegetables, leafy vegetables, seeds, fruits, and animal foods). Samples of identified items were either provided by participants (if available) or were collected by the research team; these samples were then sent for classification to a team of experts at the Botany department of Birsa Agricultural University, Ranchi. The photographed food item (around 50–100 g; all parts) was collected, wrapped in paper towels, placed in a well perforated polythene bag and sent to the botanists for identification/confirmation of the botanical classification. Subsequent to the botanical classification, the Indian Food Composition tables were checked for availability of the nutritive values of the classified foods and finally a list of the items not available in the Indian food composition tables was prepared for collection for nutrient analysis.

The food samples short listed for nutrient analysis and available at the time of survey/sampling were collected from the field site or procured from the local market (whichever was the usual mode of procurement in the community). Flesh foods were not analysed in this study.

Each of the procured samples was weighed, wrapped in clean paper towels, placed in well perforated polythene bags, and placed in a laboratory travel cooler box lined with freezer packs for transportation to the site of interim storage. Five hundred grams of each of the vegetables/fruits/green leafy vegetables collected was sent to the National Accreditation Board for Testing and Calibration Laboratories (NABL) certified laboratory for analysis.

#### Nutrient analysis

The nutrient analysis was done according to standard reference protocols. The specific methodology is listed in table 2. The parameters analyzed for the raw/uncooked samples included energy, carbohydrates, total fat, total carbohydrate, sugar, dietary fiber, vitamin A (as beta carotene), thiamine (vitamin B_1_), riboflavin (vitamin B_2_), niacin (vitamin B_3_), vitamin C, calcium, iron, zinc, sodium, and folic acid.

## RESULTS

### Traditional Diets of Oraon Tribes

The qualitative enquiry revealed that rice was the staple food for the community. Rice in the form of puffed rice and rice flakes were also commonly consumed. Meals consisted of rice with green leafy vegetables (GLVs). Pulses use was reportedbut they were not being consumed daily. Consumption of roots and tubers both cultivated and from the wild were also reported. The availability and consumption of a large variety GLVs (*saag*) was reported

**TABLE 2:** List of Parameters and Relevant Methodological Details for Nutrient Analysis

S. No.	Test parameter/Standard	Method of testing	Methodology	Reference method
1.	Energy, Kcal/100g	IFS/C/STP/FC/008	NIN	1. IS 14433: 2007 (Reaff. 2012); Clause 6.10.1 C
				2. IS 1656: 2007 (Reaff. 2012); Appx. C.
2.	Protein (*N* × 6.25), %	IS: 7219-1973	Titrimetric	
3.	Total Fat, %	IFS/C/STP/FC/012	Gravimetric	IS: 4684: 1975
4.	Total Carbohydrate, %	IFS/C/STP/FC/013	By difference	1. IS 1656-2002
				2. AOAC 19th ed. 986.25 (2010), Method E
5.	Sugar, %	IFS/C/STP/FC/010	Titrimetric	FSSAI Manual of Methods of Analysis of Food, Lab Manual 4
6.	Dietary Fiber, %	IFS/C/STP/FC/007	Kit method	Sigma Kit based on AOAC 985.29
	Vitamins			
7.	Vitamin A (as ß-carotene), mg/100 g	IFS/C/STP/LC/025	HPLC	*International Food Research Journal* 19 (2): 531–535 (2012).
8.	Vitamin B_1_, B_2_, B_3_, mg/100 g	IFS/C/STP/LC/002	HPLC	AACC.1995.86–90 and Roche Analytical Manual
9.	Vitamin C, mg/100 g	IS: 5838-1970	HPLC	
10.	Folic Acid, μg/kg	IFS/M/STP/027	ELISA	
	Minerals			
11.	Calcium, mg/100 g	IFS/C/STP/AAS/004	AAS	AOAC 999.10 and AOAC 999.11
12.	Iron, mg/100 g	IFS/C/STP/AAS/004	AAS	AOAC 999.10 and AOAC 999.11
13.	Zinc, mg/kg	IFS/C/STP/AAS/004	AAS	AOAC 999.10 and AOAC 999.11
14.	Sodium, mg/100 g	IFS/C/STP/AAS/004	AAS	AOAC 999.10 and AOAC 999.11

in all the discussions. The participants differentiated between GLV which are grown in the *bari* or foraged from the forest. Some families had small plots of land for cultivating vegetables for their own needs. Consumption of fruits especially wild fruits, seasonal fruits and flesh foods such as wild meat, birds, ant eggs, rodents, and molluscs were also reported. Hunting (during *fagun*, or March–April) activities were limited and were pursued only during the festival season. The indigenous foods (*n* = 136) identified through the FGDs are listed in table 3. Hunting for game was done only during major festivals, but smaller birds and animals were trapped by the men. Both men and women went into the forest to collect fire wood and non-timber forest products such as honey, leaves (for making plates) for selling in the market. The discussion continued with inquiries regarding their traditional diets and dietary changes due to the availability of packaged foods and market bought items.

### Food Preferences and Ranking of Local Foods

**FIGURE 1:**
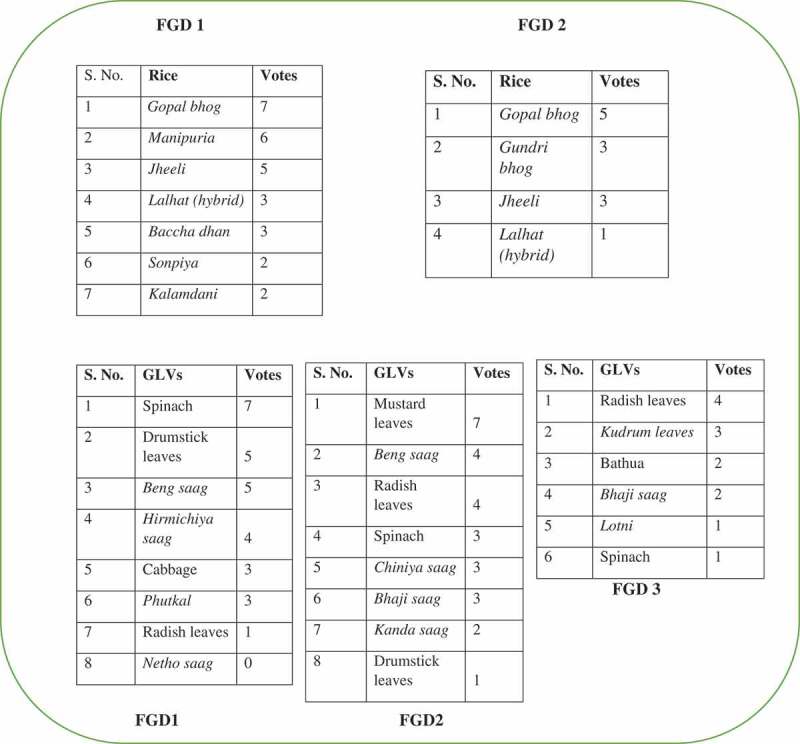
Food items listed through pairwise ranking.

Out of the foods listed, the ones commonly consumed under each food group and preferred by the community were listed through pairwise ranking. A demonstration of the outcome of pair wise ranking done through the FGDs for different food groups is given for preferred rice variety and green leafy vegetables (GLVs) in figure 1. It is important to note that though the community mostly consumed improved or high yielding varieties (HYV) of rice, when asked about their preferences in terms of taste, the majority chose indigenous varieties. In two of the study villages, indigenous rice (Oryza sativa) varieties like Gopal bhog (rice), Manipuria, Gundri and Jheeli were the most preferred varieties followed by the commonly consumed HYV called lalhat/lalat. Among the GLVs *Beng* (Centella asiatica) and *Kudrum* (Hibiscus cannabinus); *Hirmichiya* (Enhydra fluctuans), *Bathua* (Chenopodium album), *Phutkal* (Ficus geniculata), *Chiniya* (Brassica campensis pekinensis) and *Bhaji* (Amaranthus viridis) were the most preferred and consumed indigenous varieties. In case of other vegetables, roots and tubers, no indigenous variety was commonly consumed or preferred.

**FIGURE 2:**
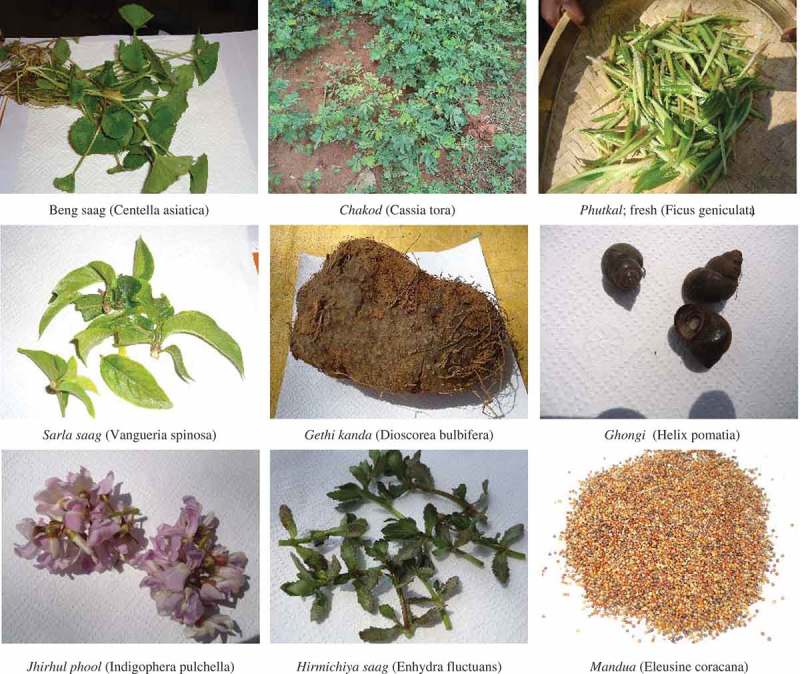
Photographs of representative food items collected.

Based on their common names in the Indian food composition table (table 4) (Gopalan et al. 1989), taxonomic classification was found to be available for 47 foods. The foods (*n* = 30) for which classification was unavailable were collected and sent for identification by an expert at the Birsa Agricultural University (Botany Department). Finally, the taxonomic classification provided was verified from other literature sources (table 5) (Gopalan et al. 1989; Kharel, Acharya, and Rai 2009; Kumari and Kumar 2001; Mehta, Negi, and Ojha 2010; Shukla et al. 2013; Sinha and Lakra 2005, 2007; Srivastava and Soreng 2012; Torkelson 1996; Tropical Forest Research Institute 2009). Some representative pictures of the food samples are provided in figure 2.

**TABLE 3:** Indigenous Foods with Edible Parts and the Place of Procurement

Name of the food item	English name	Part consumed	Accessed/Grown
*Lalhat (desi), Gopal bhog, Kalamdani, Baccha dhan, Gundri bhog, Goda dhan, Karhani, Sonpiya, Manipuriya, Jheeli, Don chawal, Khandgiri*	Rice varieties	Grain	Field
*Makka*	Maize	Grain	Field, market
*Mandua*	Finger millet	Millet	Field
*Salkaya (Gondla)*	Variety of millet	Millet	Field
*Ghongi*	Snail	Meat	Farm
*Bhawra*	Honey bee		
*Uffiya*	Variety of insect		
Baumungi/Saaras	Crane		
*Batakh*	Duck meat		
*Batakh anda*	Duck eggs		
*Demta*	Ant eggs	Meat	Farm, forest
*Tumbil*	Insect		
Moosa	Rat		
*Gilahri*	Squirrel		
*Sahi*	Porcupine		
*Barah*	Wild pig		
*Saanp*	Snake		
*Teetar, Goraiya/Gerua Tota* Mynah, *Perua, Haarla/Chidiya, Kauwa Padki, Mayur*	Birds		
*Kotara*	Deer-like animal		
*Fulchumbi, Lambha/Kulhai*	Animal		
*Bhalu*	Bear		
*Siyar*	Jackal		
*Chamkadar*	Bat	Meat	Field, farm, forest
*Budnu, Magai, Getu, Pothi, Chingri, Dungdungiya, Mugri, Jiya, Girsa, Singhi*	Varieties of fish	Meat	Farm, pond
*Setua*	Mussel	Meat	Pond
*Kenkda*	Crab		
*Kachua*	Tortoise		
*Khesari dal, Barbatti*	Varieties of legumes	Seed	Field
*Chakod, Raksaag, Kudrum, Bhaji, Sakhin saag*	Varieties of green leafy vegetables	Leaves	Weed, field, kitchen garden
*Aloo saag*	Potato greens		
*Kanda saag*	Sweet potato greens		
*Saaru/Bhoda*	Colocasia leaves	Leaves	Field, forest
*Phutkal, Koinaar, Sarla/Katai saag*	Varieties of green leafy vegetables	Leaves	Field, farm, forest
*Bamboo*	Bamboo tender shoots		
*Amad simat/Lavaiyat, Daal saag, Chench saag*	Varieties of green leafy vegetables	Leaves	Weed, field, forest
*Hirmichiya, Chimti, Beng, Netho, Siliary, Matha, Sunsuniya, Khapra saag, Botha, Dugdugiya, Gundri saag, Kaado, Rampavan, Bhadli Saag*	Varieties of green leafy vegetables	Leaves	Weed, field
*Karmi saag*	Ipomea leaves	Leaves	Field
*Gandhari saag*	Amaranth, spined		
*Lotni,Chiniya, Patjivan/Ajooba saag*	Varieties of green leafy vegetables		
*Jhirhul phool, Kanod Phool*	Varieties of vegetable	Vegetable	Field, forest
*Kohnda Phool*	Pumpkin flower	Vegetable	Field, kitchen garden
*Sanai Phool Jangali karela*	Sunhemp flower		
*Koraiya Phool, Pandan*	Kind of vegetable	Vegetable	Forest
*Kukdi*	Kind of vegetable	Vegetable	Field
*Gangia (Lava)*			
*Sem*	Field beans		
*Kalkatiya sem*	Broad beans		
*Mooli phal*	Radish pods		
*Barbatti*	Cowpea pods		
*Gethi kanda, Aaro Kanda, Sakhin kanda*	Tubers	Tuber	Field, forest
*Jaam khukhdi, Baalu khukhdi, Badhka khukhdi, Baans khukhdi, Pathiyari, Chiriya, Jaith, Chelari, Rugda/Putu*	Mushroom	Mushroom	Field, forest
*Sarai/Saal*	Kind of fruit	Fruit	Field
*Sandhana/Karai*	Bamboo fruit	Fruit	Field, farm
*Makoi*	Kind of fruit	Field, farm	
*Kusum*	Kind of fruit	Fruit	Field, farm, forest
*Bargad ka phal*	Banyan tree figs		
*Dahu*	Kind of fruit		
*Ber*	Zizyphus		
*Keyund*	Kind of fruit	Fruit	Field, farm, forest, market
*Mahua*	Mahua, ripe	Fruit	Field, forest
*Karhi*	Kind of fruit		
*Chaar*			
*Chiraunji*	Kind of fruit		
*Karaunda*	Kind of fruit	Fruit	Forest
*Bhelua*	Marking nut (kernel)		
*Kutumba*	Kind of fruit		
*Kadam*	Kind of fruit		
*Redi tel*	Rapeseed oil	Oil	Market
*Dori tel*	Mahua oil	Oil (from seed)	Home, market
*Surguja*	Niger	Oilseed	Field
*Hadiya*	Alcohol	Fermented rice preparation	Home, market
*Khajur Tadi*	Alcohol	Prepared from fresh dates	Home, market
*Mahua*	Alcohol	Prepared from mahua	Home, market

Of all the foods identified in this study, the nutritive values (based on taxonomic classification) were available for 55 foods in the Indian food composition tables (Gopalan et al. 1989). These included 8 foods from among those sent for classification to the botanist and those identified by common names as stated by participants during data collection. The nutritive value of these 8 foods along with those foods (whose classification was verified from literature and nutritive value was available in Indian food composition tables are summarized in table 6 (*n* = 55). For the rest of the foods identified but not having nutritive values available in the Indian food composition tables, samples (*n* = 8), were procured based on their availability. These were then sent for nutrient analysis to a NABL certified laboratory in New Delhi. Table 7 provides their nutritive values as reported by the laboratory.

Many indigenous GLVs for which nutritive value was available in the Indian food composition tables namely *Chakod* (Cassia tora),S*aru* (Colocasia anti-quorum), *Karmi* (Ipomea reptans), *Gandhari* (Amaranthus spinosus), and *Kudrum* leaves were seen to have high levels of beta carotene (1,980–10,512 mcgm/100 g) and calcium (110–800 mg/100 g). The iron content of *Chakod, saru, Bhaji, Lal bhaji* (Amaranthus gangeticus), *Gandhari, Khapra* (Trianthema monogyna), and *Kanda leaves* (Ipomea batata) were

**TABLE 4:** List of Indigenous Foods for which Classification was Available in the Indian Food Composition Tables

S. No.	Local name of sample collected	Common name	Genus	Species
1.	*Makka* (Cereal)	Maize, dry	Zea	Mays
2.	*Mandua* (Millet)	Ragi	Eleusine	Coracana
3.	*Salkaya* (*Gondla*) (Millet)	Samai	Panicum	Miliare
4.	*Ghongi* (Meat)	Snail, big	Pila	Globosa
5.	*Demta* (Meat and eggs)	Red ants (with eggs)	Aecophylla	Smaragdina
6.	*Moosa* (Meat)	Field rat’s meat	Rattus	Argentiventer
7.	*Perua* (Meat)	Pigeon	Columbia	Livia intermedia
8.	*Pothi* (Fish)	Puti	Burbus	Sp.
9.	*Chingri* (Crustacean)	Prawn	Penaeus	Sp.
10.	*Singhi* (Fish)	Singhi	Saccobranchus	Fossilis
11.	*Setua* (Meat)	Mussel, fresh water	Margaritifera	Margaritifera
12.	*Kenkda* (Meat)	Crab, muscle	Paratephusa	Spinigera
13.	*Kachua* (Meat)	Turtle’s meat	Melanochelys	Trijuga coronata
14	Batakh (Meat)	Duck	Anas platyrhynchos	Domesticus
15.	*Batakh anda* (Duck egg)	Egg, duck		
16.	*Barbatti* (Pulse)	Cowpea	Vigna	Catjang
17.	*Kulthi dal* (Pulse)	Horse gram	Dolichos	Biflorus
18.	*Chakod* (GLV)	Fetid cassia, fresh	Cassia	Tora
19.	*Saru saag* (GLV; fresh)	Colocasia leaves (green variety)	Colocasia	Anti-quorum
20.	*Chimti saag* (GLV)	Chimti sag	Polygonum	Plebijum
21.	*Siliary saag* (GLV)	Sinduar sag	Celosia	Argentia
22.	*Bhaji sag* (GLV)	Amaranth viridis	Amaranthus	Viridis
23.	*Lal bhaji* (GLV)	Amaranth, tender	Amaranthus	Gangeticus
24.	*Aloo saag* (GLV)	Potato leaves	Solanum	Tuberosum
25.	*Matha sag* (GLV)	Mata sag (lupu)	Antidesma	Diandrum
26.	*Sarla/Katai saag* (GLV)	Sarli sag	Vangueria	Spinosa
27.	*Sunsuniya* saag (GLV)	Susni sag	Marsilea	Minuta
28.	*Karmi saag* (GLV)	Ipomea leaves	Ipomea	Reptans
29.	*Gandhari saag* (GLV)	Amaranth, spined	Amaranthus	Spinosus
30.	*Kudrum leaves/Thepa saag* (GLV)	Gogu	Hibiscus	Cannabinus
31.	*Khapra sag* (GLV)	Saravallai keerai	Trianthema	Monogyna
32.	*Kanda sag* (GLV)	Sweet potato greens	Ipomoes	Batatas
33.	*Bathua saag* (GLV; fresh)	Bathua	Chenopodium	Album
34.	*Bambu* (GLV)	Bamboo, tender shoots	Bambusa	Arundinacea
35.	*Munga saag* (GLV)	Drumstick leaves	Moringa	Oleifera
36.	*Munga phool* (Vegetable)	Drumstick flowers	Moringa	Oleifera
37.	*Kohnda Phool* (Vegetable)	Pumpkin flowers	Cucurbita	Maxima
38.	*Sanai Phool* (Vegetable)	Sanhemp flowers	Crotalaria	Juncea
39.	*Sem* (Vegetable)	Field beans, tender	Dolichos	Lablab
40.	*Bamboo/Sandhna/Karai* (Fruit)	Bamboo fruit	Bambusa	Arundinacea
41.	*Kusum* (Fruit)	Kusum fruit	Schleichera	Trijunga
42.	*Kendu* (Fruit)	Tumki	Diospyros	Melanoxylon
43.	*Dahu* (Fruit)	Lakooch, raw	Artocarpus	Lakoocha
44.	*Bargad ka phal* (Fruit)	Banyan tree figs	Ficus	Bengalensis
45.	*Karaunda* (Fruit)	Karonda, fresh	Carissa	Carandas
46.	*Bhelua* (Fruit)	Marking nut (kernel)	Semecarpus	Anacardium
47.	*Surguja* (Oilseed)	Niger seeds	Guizotia	Abyssinica

**TABLE 5:** List of Indigenous Foods Collected and Sent for Classification along with Verification from Other Sources of Literature

		Identification done by the botanist		
S. No.	Local name of sample collected	Genus	Species	Verification from other sources
1.	*Nanhiya* (Rice)	Oryza	Sativa	−
2.	*Lalhat* (Rice)	Oryza	Sativa	−
3.	*Gopal Bhog* (Rice)	Oryza	Sativa	−
4.	*Jheeli Dhan* (Rice)	Oryza	Sativa	−
5.	*Gondri Bhog* (Rice)	Oryza	Sativa	−
6.	*Mahto Dhan* (Rice)	Oryza	Sativa	−
7.	*Kapur Bhog* (Rice)	Oryza	Sativa	−
8.	*Phutkal* (GLV; fresh)	Ficus	Geniculata	Ficus geniculata (Shukla et al. 2013)
9.	*Lotni saag* (GLV; fresh)	Brassica	Juncea	−
10.	*Hirmichiya saag* (GLV; fresh)	Enhydra	Fluctuans	Limnophila conferta Benth. (Scrophulariaceae) (Sinha and Lakra 2007)
11.	*Beng saag* (GLV; fresh)	Centella	Asiatica	Centella asiatica Linn. (Sinha and Lakra 2007)
12.	*Netho saag* (GLV; fresh)	Oxalis	Corniculata	Oxalis corniculata Linn. (Geraniaceae) (Sinha and Lakra 2007)
13.	*Koinaar saag* (GLV; fresh)	Bauhinia	Purpurea	Bauhinia purpurea (Gopalan et al. 1989)
14.	*Chiniya saag* (GLV; fresh)	Brassica	Campestris pekinensis	Brassica pekinensis
15.	*Patjivan/Ajooba* (GLV; fresh)	Bryophyllum	Spp.	Bryophyllum pinnatum (Kumari and Kumar 2001)
16.	*Amad Simat/Lavaiyat* (GLV; fresh)	Medicago	Lupulina	Medicago lupulina (Sinha and Lakra 2007)
17.	*Gundri saag* (GLV; fresh)	Alternanthera	Sessilis	Alternanthera sessilis Linn. (Srivastava and Soreng 2012)
18.	*Barbatti/Boro* (Vegetable)	Vigna	Sesquipedalis	Vigna catjang (Common name **–** Cowpea pods) (Gopalan et al, 1989)
19.	*Rugra/Puttu* (Vegetable)	Geastrum	Spp.	Geastrum (Common name- *Rugra, Putu*) (Srivastava and Soreng 2012)
20.	*Jaam Khukhdi* (Mushroom)	Boletus	Edulis	Boletus edulis (Common name **–***Jamun khukhri*) (Srivastava and Soreng 2012)
21.	*Jhirhul phool* (Vegetable)	Indigofera	Pulchella/Cassioides	Indigo pulchella Roxb. (Common name– *Jerhul*) (Sinha and Lakra 2005)
22.	*Kalkatiya sem* (Vegetable)	Vicia	Faba	Vicia faba (Common name – Broad beans) (Gopalan et al. 1989)
23.	*Mooli Phal* (Vegetable)	Raphanus	Sativus	−
24.	*Jangali Karela* (Vegetable)	Momordica	Dioicia	Momordica dioicia (Common name – *Kankoda*) (Gopalan et al. 1989)
25.	*Gethi Kanda* (Tuber)	Dioscorea	Bulbifera	Dioscorea bulbifera Linn. (Dioscoreaceae) (Torkelson 1996)
26.	*Sakhin Kanda* (Tuber)	A species of colocasia		
27.	*Mahua* (Fruit)	Madhuca	Latifolia	Bassia longifolia (Common name – Mahua, ripe) (Gopalan et al. 1989)
28.	*Kutumba* (Fruit)	Solanum	Indicum	Solanum indica Linn. (Sinha and Lakra 2007)
29.	*Ber* (Fruit)	Zizyphus	Jujube	Zizyphus jujuba (Common name – Zizyphus) (Gopalan et al. 1989)
30.	*Lahsun saag*	Allium	Sativum	Allium sativum (Mehta et al. 2010)

in the range of 3.49 to 38.5 mg/100 g. The vitamin C content of GLVs like *Chakod, Saru, Karmi, Gandhari, Kudrum, Khapra, kanda* leaves, were high (12 to 82 mg/100 g). *Bhaji* was found to have exceptionally high vitamin C content (179 mg/100 g). The GLVs *Lotni*, (Brassica juncea), *Hirmichiya, Chiniya, Lahsun* (Allium sativum) were found to be rich sources of calcium (mg) (range 221 to 389 mg/100 g of edible portion), iron (mg) (range 5.95 to 19.77 mg/100 g) and beta carotene (980 to 5,100 mcgm/100 g). *Beng saag* (Centella asiatica) was found to be exceptionally rich in iron (55.66 mg/100 g) and dietary fiber (7.5 mg/100 g). *Mandua* (Eleusine coracana), a millet was found to have high levels of calcium (344 mg/100 g) and dietary fiber (11.5 mg/100 g). The pulses consumed, namely *Khesari dal* (Lathyrus sativus) and *Barbatti* (Vigna catjang), were rich sources of thiamine (0.39 and 0.51 mg/100 g, respectively) and iron (6.3 and 8.6 mg/100 g, respectively). The indigenous rice variety, *Lalhat desi* was found to be high in folic acid (14.09 mcgm/100 g). A dried variety of GLV, *Phutkal* was also analyzed and found to be rich in calcium (672 mg/100 g), iron (8.89 mg/100 g), zinc (4.63 mg/100 g), and dietary fiber (45.1 mg/100 g).

Flesh foods like snail and fresh water mussel, *Singhi* fish (Saccobranchus fossilis), and crab consumed by the community were seen to be rich sources of protein and calcium (592–1,370 mg/100 g).

**TABLE 6:** List of Food Items with their Nutritive Value Available in the Indian Food Composition Tables

S. No.	Parameter/ Food items	Common name (Gopalan et al. 1989)	Energy (Kcal/100 g)	Protein (g/100 g)	Total fat (g/100 g)	Total carbo-hydrate (g/100 g)	Crude fiber (g/100 g)	Dietary fiber (g/100 g)	Vitamin A (ß-carotene) (μg/100 g)	Vit B_1_ (mg/100 g)	Vit B_2_ (mg/100 g)	Vit B_3_ (mg/100 g)	Vit C (mg/100 g)	Calcium (mg/100 g)	Iron (mg/100g)	Zinc (mg/100g)	Sodium (mg/100 g)	Folic acid (μg/100 g) (Total)
1.	Rice, raw milled (Cereal)	Rice, raw milled, 5%	345	6.8	0.5	78.2	0.2	4.1	0	0.06	0.06	1.9	0	10	0.7	1.4		8.0
2.	*Makka* (Cereal)	Maize, dry	342	11.1	3.6	66.2	2.7	11.9	90	0.42	0.10	1.8	0	10	2.3	2.8	15.9	20.0
3.	*Mandua* (Millet)	*Ragi*, Finger millet	328	7.3	1.3	72.0	3.6	11.5	42	0.42	0.19	1.1	0	344	3.9	2.3	11.0	18.3
4.	*Salkaya* (*Gondla*) (Millet)	*Samai*	341	7.7	4.7	67.0	7.6		0	0.30	0.09	3.2	0	17	9.3	3.7	8.1	9.0
5.	*Ghongi* (Meat)	Snail, big	97	10.5	0.6	12.4								870				
6.	*Demta* (Meat and eggs)	Red ants with eggs	131	13.4	4.6	9.1	−							104				
7.	*Moosa* (Meat)	Field rat’s meat	104	23.6	1.0	0.1								30				
8.	*Perua* (Meat)	Pigeon	137	23.3	4.9									12				
9.	*Pothi* (Fish)	*Puti*	106	18.1	2.4	3.1						0.3	15	110	1.0			
10.	*Chingri* (Fish)	Prawn	89	19.1	1.0	0.8			0	0.01	0.10	4.8		323	5.3		66.0	
11.	*Singhi* (Fish)	*Singhi*	124	22.8	0.6	6.9						0.8		670	2.3		53.0	
12.	*Setua* (Meat)	Mussel, fresh water	81	14.5	1.6	2.1								592				
13.	*Kenkda* (Meat)	Crab, muscle	59	8.9	1.1	3.3			780^f^			3.1		1,370	21.2			
14.	*Kachua* (Meat)	Turtle’s meat	86	16.5	1.5	1.5								7				
15.	*Batakh* (Meat)	Duck	130	21.6	4.8	0.1								4				
16.	*Batakh anda* (Duck egg)	Egg, duck	181	13.5	13.70	0.8			405^f^	0.12	0.26	0.2		70	2.5			80.0
17.	*Barbatti* (Pulse)	Cowpea	323	24.1	1.0	54.5	3.8		12	0.51	0.20	1.3	0	77	8.6	4.6	23.2	133.0
18.	*Kulthi dal* (Pulse)	Horsegram, whole	321	22.0	0.5	57.2	5.3		71	0.42	0.20	1.5	1	287	6.77	2.8	11.5	
19.	*Chakod* (GLV)	Fetid cassia, fresh	49	5.0	0.8	5.5	2.1		10,512	0.08	0.19	0.8	82	520	12.4			
20.	*Saaru saag* (GLV; fresh)	Colocasia leaves (green variety)	56	3.9	1.5	6.8	2.9	6.6	5,920	0.22	0.26	1.1	12	227	10.0			
21.	*Chimti saag* (GLV)	*Chimti sag*	46	3.2	0.7	6.9	2.1							194				
22.	*Koinaar saag* (GLV)	*Konar sag*	62	3.6	1.0	9.7	5.5							312				
23.	*Siliary saag* (GLV)	*Sinduar sag*	38	2.0	0.7	5.8	1.5							323				
24.	*Bhaji sag* (GLV)	Amaranth viridis	38	5.2	0.3	3.8	6.1						179	330	18.7			
25.	*Lal bhaji* (GLV)	Amaranth, tender	45	4.0	0.5	6.1	1.0	4.0	5,520	0.03	0.30	1.2	99	397	3.49	0.18	230.0	149.0
26.	*Aloo saag* (GLV)	Potato leaves	40	4.4	0.9	3.6	1.3							120				
27.	*Matha saag* (GLV)	*Mata sag (lupu)*	303	7.2	4.8	57.8	13.5							1,717				
28.	*Sarla/Katai saag* (GLV)	*Sarli sag*	86	4.0	1.1	14.9	1.5							127	−			
29.	*Sunsuniya saag* (GLV)	*Susni sag*	46	3.7	1.4	4.6	1.3							53	−			
30.	*Karmi saag* (GLV)	Ipomea leaves	28	2.9	0.4	3.1	1.2		1,980	0.05	0.13	0.6	37	110	3.9			
31.	*Gandharisaag* (GLV)	Amaranth, spinosus	43	3.0	0.3	7.0	1.1		3,564	0			33	800	22.9			
32.	*Kudrum leaves/Thepa saag* (GLV)	*Gogu*	56	1.7	1.1	9.9		3.8	6,970	0.07	0.39	1.1	20	172	2.28	0.27		
33.	*Khapra sag* (GLV)	*Saravallai keerai*	24	2.0	0.4	3.2	0.9						70	100	38.5			
34.	*Kanda sag* (GLV)	Sweet potato greens	63	4.2	0.8	9.7	2.4		750	0.07	0.24	1.7	27	360	10.0			
35.	*Bathua saag* (GLV)	Bathua	30	3.7	0.4	2.9	0.8		1,740	0.01	0.14	0.6	35	150	4.2			
36.	*Bambu* (Vegetable)	Bamboo, tender shoots	43	3.9	0.5	5.7			0	0.08	0.19	0.2	5	20	0.1			
37.	*Munga saag* (GLV)	Drumstick leaves	92	6.7	1.7	12.5	0.9		6,780	0.06	0.05	0.8	220	440	0.85	0.16		
38.	*Gundri saag* (GLV)	Ponnanganni	73	5.0	0.7	11.6	2.8		1926	0	0.14	1.2	17	510	1.63			
39.	*Munga phal* (Vegetable)	Drumstick flowers	50	3.6	0.8	7.1	1.3							51				
40.	*Kohnda Phool* (Vegetable)	Pumpkin flowers	39	2.2	0.8	5.8	0.7							120				
41.	*Sanai Phool* (Vegetable)	Sanhemp flowers	66	4.8	0.6	10.4	3.9							200				
42.	*Sem* (Vegetable)	Field beans, tender	48	3.8	0.7	6.7	1.8		187	0.10	0.06	0.7	9	210	0.83	0.40	55.4	
43.	*Kalkatiya sem* (Vegetable)	Broad beans	48	4.5	0.1	7.2	2.0	8.9	9	0.08		0.8	12	50	1.4		43.5	
44.	*Jangali karela* (Vegetable)	*Kankoda*	52	3.1	1.0	7.7	3.0		1,620	0.05	0.18	0.6		33	4.6			
45.	*Barbatti* (Vegetable)	Cowpea pods	48	3.5	0.2	8.1	2.0		564	0.07	0.09	0.9	14	72	2.5			
46.	*Bamboo/Sandhna/ Karai* (Fruit)	Bamboo fruit	153	3.9	0.1	34.2	3.9		11	0.09	0.09		1	10	1.5			
47.	*Kusum* (Fruit)	Kusum fruit	53	1.5	0.8	9.9	0.6							15				
48.	*Kendu* (Fruit)	*Tumki*	112	0.8	0.2	26.8	0.8		361	0.01	0.04	2.3	1	60	0.5			
49.	*Dahu* (Fruit)	*Lakuch*	66	0.7	1.1	13.3	2.8		254	0.02	0.15	0.3	135	50	0.5			
50.	*Mahua* (Fruit)	Mahua, ripe	111	1.4	1.6	22.7	−		307	−	−	−	40	45	0.23			
51.	*Bargad kaphal* (Fruit)	Banyan tree figs	72	1.7	2.0	11.8	8.5							364				
52.	*Karaunda* (Fruit)	*Karonda*, fresh	42	1.1	2.9	2.9	1.5							21				
53.	*Bhelua* (Fruit)	Marking nut (kernel)	587	26.4	36.4	28.4	1.4							295	6.1			
54.	*Ber* (Fruit)	Zizyphus	74	0.8	0.3	17.0		3.8	21	0.02	0.05	0.7	76	4	0.50	0.10		
55.	*Surguja* (Oilseed)	Niger seeds	515	23.9	39.0	17.1	10.9			0.07	0.97	8.4		300	56.7			

^f^Values represent microgram of vitamin A.

**TABLE 7:** Nutritive Values of Foods Analyzed at Laboratory

S. No.	Parameter/Food items	Energy (Kcal/100 g)	Protein (g/100 g)	Total fat (g/100 g)	Total carbo-hydrate (g/100 g)	Sugar (g/100g)	Dietary fiber (g/100 g)	Vitamin A (ß-carotene) (μg/100 g)	Vit B_1*_ (mg/100 g)	Vit B_2*_ (mg/100 g)	Vit B_3_ (mg/100 g)	Vit C (mg/100 g)	Calcium (mg/100 g)	Iron (mg/100 g)	Zinc (mg/100 g)	Sodium (mg/100 g)	Folic acid (μg/100 g)
1.	*Lalhat, desi* (Rice)	363	6.6	1.6	80.6	ND	3.9	ND	ND	ND	ND	3	14	1.44	0.81	5.5	14.09
2.	*Lotni saag* (GLV)	28	2.2	ND	4.3	ND	3.3	1,750	0.8	ND	10.5	4	389	19.77	0.79	1.3	3.2
3.	*Hirmichiya saag* (GLV)	38	2.1	ND	6.5	ND	4.4	980	0.96	ND	ND	4	246	16.99	0.94	80.0	9.6
4.	*Beng saag* (GLV)	54	1.9	ND	11.0	ND	7.5	500	0.53	ND	ND	5	231	55.66	1.92	5.2	10.5
5.	*Chiniya saag* (GLV)	31	1.5	ND	6.3	ND	3.5	4,290	ND	0.33	27.3	11	274	5.93	ND	55.9	0.96
6.	*Lahsun saag* (GLV)	34	3.1	ND	5.4	ND	4.9	5,100	ND	ND	**	6	221	5.95	0.21	10.8	2.9
7.	*Phutkal saag*, dried (GLV)	324	18.7	1.8	58.4	ND	45.1	530	ND	ND	ND	5	672	8.89	4.63	11.3	10.9
8.	*Gethi kanda* (Tuber)	95	2.4	ND	21.1	ND	3.5	30	ND	ND	ND	4	20	4.09	0.38	0.08	2.8

*Note.* ND = Not detected.

*The lab method had an (level of detection) LOD of 0.3 mg/100 g for vitamin B1 and vitamin B2. These values are developed based on rich sources of these vitamins. Hence for most of the foods identified, the laboratory-tested value for these vitamins has been declared as ND.

**Insufficient sample.

### Medicinal Uses of Indigenous Plants

Indigenous plants used for medicinal purposes were identified by the FGD participants. In general, majority of the plants were useful for easing stomach ailments, management of pain and fever and in improving overall health.

*Beng saag* was the most versatile medicinal plant identified by the participants and was highly preferred among the GLVs as well. *Beng* was reported to ease stomach ailments and jaundice, maintain blood pressure and keep the heart healthy, reduce blood sugar, and improve mental capacity.

The leaves of the *tulsi* (Ocimum tenuiflorum), *katai* (Vangueria spinosa), tamarind (Tamarindus indica), *phutkal,* and the fruit of the *sarai* (Bambusa arundinacea) plant were used to treat stomach ailments such as diarrhea, and vomiting.

GLVs such as *beng, kulthi* (Dolichos biflorus), *neem* (Azadilrachta indica), and the bark of the *charaigodwa* (Vitex peducularis) were believed to improve immunity and induce healing. The participants explained that these plants increased one’s appetite and kept the body cool. *Beng, charaigodwa,* and *munga* (Moringa oleifera) were believed to maintain blood pressure.

The tuber *kariya haldi* (Curcuma caesia) or the rare black turmeric was used for treating chest pains. *Bhuineem* (Andrographis paniculata) leaves were considered blood purifiers and were used for fevers or malaria. *Papaya* leaves (Carica papaya) were also used to cure fever in children.

The seeds of the *chakod* plant were used to treat tuberculosis. The participants identified the use of *koraiya* bark (Holarrhena antidysenterica) as a local treatment for heat stroke. They mix *koraiya* with *hadiya* or fermented rice wine. *Charaigodwa* and *mahua* fruit were used to relieve pain and swelling. *Katai* leaves could be applied to cuts.

Barks from trees (*bakla*) or seeds were boiled or grounded and consumed as tea for deriving their medicinal use. These plants were mostly cultivated in the *bari* or foraged from the forests. Freshly prepared GLVs were used for most of the medicinal uses, with the exception of the tamarind and neem leaves, which were dried and then later consumed.

## DISCUSSION

Our study demonstrates a wide diversity of indigenous foods available and consumed from the natural environment of the Oraon tribes. Specifically, a number of micronutrient rich plant foods were part of their daily dietary intake. The preferred and commonly consumed food items identified through pairwise ranking were particularly rich in iron, calcium, vitamin A, vitamin C and folate (Gopalan et al. 1989). The food items which were analyzed in the laboratory as part of the study were also found to be good sources of one or more micronutrients.

Some of the indigenous foods identified in the study also reportedly had medicinal properties which was known to the local community based on practical knowledge and traditional wisdom. Based on some of our findings and evidence from previous literature, there is likely a scientific basis to these beliefs. For example, the nutrient analysis of *beng saag* revealed a high iron and dietary fiber content. Its medicinal properties are believed to be due to its triterpenoid and saponin content (Gohil, Patel, and Gajjar 2010). *Phutkal* has high levels of zinc which may be the reason for its efficacy in treating diarrhea. The local environment of the Oraon community thus presented a rich and ready source of indigenous plants that were used for maintaining good health and treating illnesses. The women foraged for these plants from nearby forest areas and also cultivated them in their kitchen gardens or plots. Access to land (either communal area or kitchen gardens) will help protect this treasure trove of traditional knowledge and sustain biodiversity. Some villages inhabited by the Oraon are in difficult terrain and remote areas; use of this indigenous knowledge about medicinal benefits may potentially be used as a first line of management for minor ailments before accessing standard health care. Supporting and advocating for the consumption of indigenous plants for their medicinal properties through local policy interventions or knowledge and behavior change communication could present opportunities for improving community health outcomes.

While studies have documented the various uses and immense diversity of the flora and fauna in Jharkhand (Pradhan, Mishra, and Mohapatra 2011; Sinha and Lakra 2007) and some efforts are underway to conserve the natural biodiversity for food and livelihood security (Gene Campaign 2014), the analysis and documentation of nutrient content of these and their preferential consumption is rare. Our findings are significant given that the community in question has been reported to have high rates of chronic malnourishment in all population groups (Das and Bose 2012) which compromises the health and well-being of women and children in particular and also the community at large. Multiple government supported efforts are trying to tackle the prevalence of undernutrition and micronutrient deficiency (MoHFW 2014; MWD 2014) . However, the rates of progress are slow and the communities are often dispersed in geographically difficult terrain. The enormous natural diversity present in the indigenous foods with the potential to contribute to nutritionally complete dietary patterns, the existence of transgenerational knowledge of their uses within the community and the ease of assimilation of these foods into the routine diets of the tribals can be leveraged to address malnutrition. The local communities also employ many conservation strategies to maintain these food resources. Support and advocacy for their increased consumption can be an important supplementary strategy to improve nutritional status within this tribal group. Listing and identification of these foods could also be a way of identifying underutilized items and advocating their incorporation into the diet (Kuhnlein and Turner 1996). In the context of promoting consumption of indigenous foods for improved nutrition, kitchen gardens for cultivating these nutrient rich foods can be an effective strategies. Use of indigenous knowledge can be also be leveraged for procuring and utilization of these foods from nearby forests and the like. This information can be further incorporated into nutrition education materials at a community level for their effective dissemination (Food and Agriculture Organization 1993). In addition, the promotion of the continued use of these foods in the diet of the whole family rather than shifting to more “modern” diets will prevent the advance of the dual burden of malnutrition that is being observed across most developing economies (Dixit et al. 2011).

Research into indigenous foods and the nutritional practices related to these foods are gaining momentum for many reasons. Firstly, the scope of these foods to provide a nutrient rich diet by virtue of their diversity is considered important for maintaining a holistic health status through natural means. Secondly, the propagation of the consumption of these foods provides a buffer against the increasing displacement of traditional dietary patterns by marketed, processed foods. Though the Food and Agricultural Organization has been involved in the support and promotion of knowledge regarding indigenous foods across the Americas and Africa, data in the form of a comprehensive biorepository of indigenous foods is very limited in India (Bhattacharjee et al. 2009). While, we have discussed the nutritional aspects of the foods identified, an effort to appraise their true potential for providing improved nutritional security would be a desirable activity to obtain information on the contribution of these foods in the daily diet.

## CONCLUSION

Our study is step towards documenting the nutrient rich indigenous foods in this tribal community which could be used for quantification of nutrient intake in this community. To the best of our knowledge, no previous study has looked at the anthropological, dietary and nutritional aspects of the indigenous foods in the tribals of Jharkhand in an integrated manner. We would also like to highlight the immense scope of further study in this geographical area with such tremendous environmental biodiversity. Data on these aspects could be a repository of information for botanists, agriculturists and nutritional experts alike and form a valuable resource for researchers and for the community to build upon and preserve.

## ACKNOWLEDGMENTS

The authors acknowledge the contribution of all Oraon respondents in the study villages for sharing their knowledge about their rich heritage of indigenous and traditional foods. They thank Professor R. P. Singh “Ratan” and his team from Birsa Agricultural University for carrying out the taxonomic classification of the food samples. They also acknowledge the contribution of Ms. Keya Chatterjee, Mr. Alok Kundu, and their team at “Ekjut,” a local NGO, for providing support for data collection and facilitating an excellent team of fieldworkers; thank all the field staff, and anganwadi workers in the study villages, for their support; and acknowledge the contribution of Intertek Laboratories in analyzing the food samples.

## FUNDING

This study was funded by the Wellcome Trust Capacity Strengthening Strategic Award to the Public Health Foundation of India and consortium of UK universities.
